# Distributed Network, Wireless and Cloud Computing Enabled 3-D Ultrasound; a New Medical Technology Paradigm

**DOI:** 10.1371/journal.pone.0007974

**Published:** 2009-11-19

**Authors:** Arie Meir, Boris Rubinsky

**Affiliations:** 1 Center for Bioengineering in the Service of Humanity and Society, School of Computer Science and Engineering, Hebrew University of Jerusalem, Jerusalem, Israel; 2 Graduate Program in Biophysics, Department of Mechanical Engineering, University of California, Berkeley, California, United States of America; The University of Queensland, Australia

## Abstract

Medical technologies are indispensable to modern medicine. However, they have become exceedingly expensive and complex and are not available to the economically disadvantaged majority of the world population in underdeveloped as well as developed parts of the world. For example, according to the World Health Organization about two thirds of the world population does not have access to medical imaging. In this paper we introduce a new medical technology paradigm centered on wireless technology and cloud computing that was designed to overcome the problems of increasing health technology costs. We demonstrate the value of the concept with an example; the design of a wireless, distributed network and central (cloud) computing enabled three-dimensional (3-D) ultrasound system. Specifically, we demonstrate the feasibility of producing a 3-D *high end* ultrasound scan at a central computing facility using the raw data acquired at the remote patient site with an inexpensive *low end* ultrasound transducer designed for 2-D, through a mobile device and wireless connection link between them. Producing high-end 3D ultrasound images with simple low-end transducers reduces the cost of imaging by orders of magnitude. It also removes the requirement of having a highly trained imaging expert at the patient site, since the need for hand-eye coordination and the ability to reconstruct a 3-D mental image from 2-D scans, which is a necessity for high quality ultrasound imaging, is eliminated. This could enable relatively untrained medical workers in developing nations to administer imaging and a more accurate diagnosis, effectively saving the lives of people.

## Introduction

During the last century, major advances in medical technology have led to substantial improvements in health care. This has come at a cost; the health care technology has become complex and expensive which, in turn, has led to a very wide disparity in health care delivery between those who have the financial resources to benefit from the advanced medical technology and those that do not. The ultimate outcome of this situation is that the majority of the world population does not have access to advanced medical technology and advanced health care. For instance, according to WHO reports, “Around 95% of medical technology in developing countries is imported, much of which does not meet the needs of national health care systems. Over 50% of equipment is not being used, either because of a lack of maintenance or spare parts, because it is too sophisticated or in disrepair, or simply because the health personnel do not know how to use it.” [1]. This situation is particularly acute in the field of medical imaging, which is required for correct diagnostic in about 20% to 30% of cases worldwide and which is not available to over 60% of the world population [2]. The challenges in diagnostic imaging in developing countries include: a severe lack of safe and appropriate diagnostic imaging services because of the cost and complexity of the devices as well as a severe lack of technical skills and trained radiographers/technologists leading to a large number of images being misread or of poor quality and therefore of no diagnostic use [3].

For over a decade, our group has been working on trying to find solutions to the medical technology delivery disparity between those who have the financial resources to purchase and use these technologies and those who do not. We have identified that one major factor affecting the cost and the complexity of advanced medical technologies, such as medical imaging, is the hardware and software for data processing. Currently, medical devices are mostly stand-alone units, with redundant and practically limited computational parts, both software and hardware. The computational part becomes increasingly complex and expensive with an increase in the sophistication of the technology. In the recent years, advances in computer science, telecommunication and the Internet made information technology available at low cost to even remote villages everywhere in the world. Inspired by this fact, we conceived of a similar concept for delivering advanced medical care and medical technology. The key concept is that the computational part (hardware and software) is at a central facility, now called “cloud” which does the data processing and provides the most advanced computational service, at any time, to an unlimited number of users, connected through telecommunication to the central processing facility. The devices at the user site have limited or no data processing facility and are used primarily to transfer the raw data to the central processing facility and to display the processed data. To focus ideas, the remote devices become a dumb terminal for a central computational facility. This removes the cost and limitations of the computation, manipulation and interpretation of data from the vicinity of the patient and uses instead a central and effectively unlimited computational facility. In the vicinity of the patient only the components that directly interact with the patient and which acquire or use the raw data are needed. It should be emphasized that this is different from conventional telemedicine in which the data processing is still done in the vicinity of the patient and the processed images, for example, are sent on. In our concept the majority of the processing is done at the central facility that can be at a completely different geographical location than the patient. The central facility serves a large number of remote users and the telecommunication is used to transfer the raw or minimally processed data to this central processing.

We have demonstrated the feasibility of the concept described above using the Internet and land telecommunication for imaging with electrical impedance tomography (EIT) and for EIT monitored minimally invasive surgery [4], [5]. We have also shown that this concept can be used with cellular phone based wireless technology for remote medical imaging with EIT and that it is valuable to other computationally expensive procedures, such as developing classifiers and data bases for medical data analysis [6], [7], [8]. A review of some of the aspects of our cellular phone based work can be found in a recent Nature news feature [9].

The goal of this study is to elaborate on the fundamental paradigm we developed earlier and to illustrate the value of this paradigm with a new implementation, which could be immediately useful for medical imaging in economically disadvantaged parts of the world. We believe that in addition to EIT, ultrasound is one of the imaging modalities with the best potential to become widely used with this paradigm, due to its relatively small physical dimensions and relatively low-cost. Conventional ultrasound produces a two dimensional image. Successful use of ultrasound relies heavily on understanding the significance of the image displayed and optimal placement of the transducer through hand-eye coordination. The highly trained and experienced users of ultrasound have had to develop hand-eye coordination skills which enable them to create the mental 3D picture of the human body while watching 2D images acquired by the ultrasound system in real-time. They know exactly how to position the ultrasound (US) probe, at what angle to scan and how fast to move it along the patient's body to get a good image. Since medical personnel with such skill-sets are scarce in economically disadvantaged parts of the world, medical imaging is usually not done. In cases when medical imaging is performed the patients may be subject to wrong diagnosis and ultimately wrong treatment or no treatment at all. Three dimensional ultrasound image reconstruction, which is a relative recent addition to ultrasound, removes the need for high quality radiological expertise by allowing the physician to perform the scan without getting into the minute details of the data acquisition process such as the precise probe angle and position [10]. The challenge with industrial 3D ultrasound systems is their prohibitive cost which precludes them from being used in the developing nations, the place they are needed the most. Even in developed countries, small clinics that lack highly trained specialists, which could benefit from owning a 3D-US system, cannot afford purchasing it due to the high market price.

In this work we took the concept of processing at a central facility a step forward by implementing a fully functional 3D ultrasound system in which the 2-D intended raw data acquired at the patient site by a medical untrained person is transferred through telecommunication to a central processing facility, where it can be processed into a 3-D image or, in fact, for any conceivable use. The 3-D processed data can then be made available through communication to the data acquisition site or to an expert at any other location. The idea of coupling an ultrasound device with a communication device such as Wi-Fi adapter or a cellular phone is not new. In [11] Martini et al. have focused on the possibility of utilizing 3 g/WiFi networks for the purpose of video-streaming the acquired and processed ultrasound imaging data to the remote expert station. In [12] Dickson has evaluated several wireless communication options for ultrasound systems focusing on video-streaming capabilities in his analysis. However, to the best of our knowledge no other work has evaluated the feasibility of using telecommunication and wireless technology to transmit *raw ultrasound* data for processing on the central processing station that serves a large number of users and generates 3D image from the *raw data*.

We believe that the work presented in this study illustrates the value of our paradigm in a meaningful way. The powerful central processing facility, which can serve unlimited numbers of remote users, allows a remote unskilled user to employ an inexpensive technology and nevertheless obtain a state of the art product in terms of a 3-D ultrasound image, at a fraction of the cost and without the need for complex data processing facilities and software at the user site.

## Results

**Figure 1:**
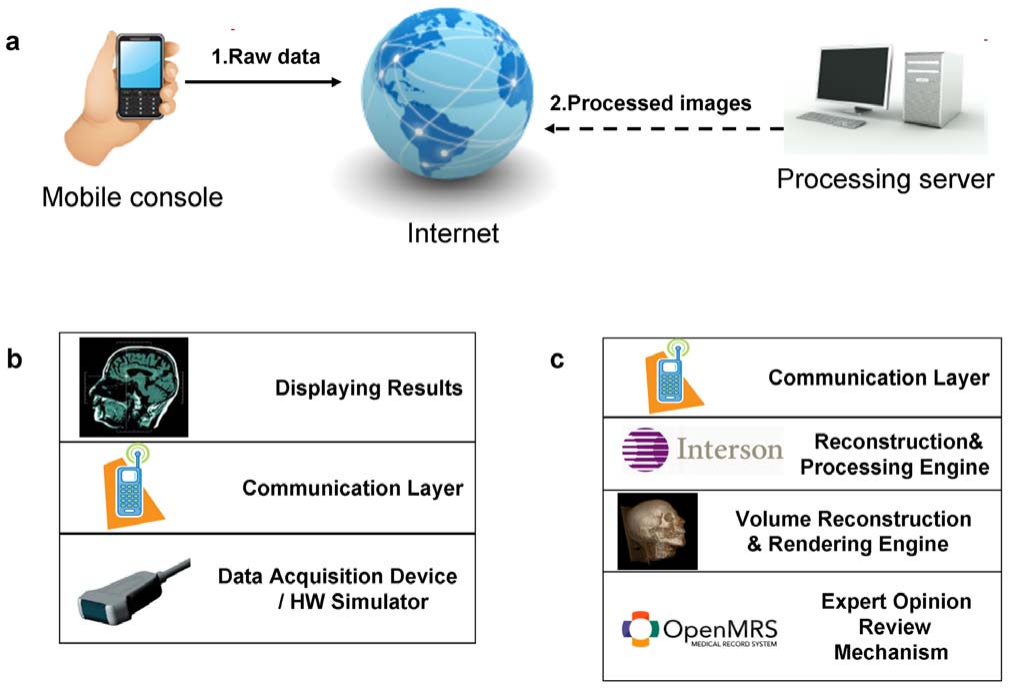
System Architecture. (a) Overall system architecture includes the mobile console component and the remote processing server (Expert System) which performs the computation-extensive work. (b) Mobile Console Architecture. The console has one or more data acquisition devices, a communication module and a display capability. (c) Server Architecture. Contains a communication module, a processing engine, a visualization engine and an expert assessment mechanism.

The system architecture aligned with the proposed general paradigm is shown to contain two major components: Mobile Console and Remote Expert System (Fig. 1a). The mobile console with its sensors acts as the data acquisition device which collects the raw data from the patient, and sends it to the remote server for processing. The processing server is capable of transforming the large amount of otherwise meaningless measurements into a human understandable form such as an image or diagnosis.

The mobile console (Fig. 1b) contains a hardware data acquisition device, a display and a communication component able to send raw data and receive results. The Processing Server (Fig. 1c) contains a communication component to receive the raw data, a processing (reconstruction) component to process the data into a useful form and a visualization (rendering) engine which shapes the data in a visually meaningful way. Optionally the server side can contain a human-assessment mechanism, which enables an expert doctor to review the results before sending them back to the mobile console.

The implementation of the general paradigm of Fig. 1 for 3-D ultrasound is given in detail in the Materials and Methods section. Specifically, we have used Lenovo R61 1.5GHz, 2GB RAM Windows XP as our server test bed running the server-side of the application software including the processing engine and OpenMRS server. For the purpose of this study we've focused primarily on a data flow in a typical obstetrics US scan, performed in B-Mode, with spatial resolution of 256×256, maintaining a contrast resolution of 8 bits (256 shades of gray). In such a study, the raw data required for the reconstruction is acquired by driving the transducer in a rectilinear, uniform direction with constant speed [13], [14]. The number of slices acquired depends on the specific application. We've used 80 slices in our study. We've used a standard, very inexpensive 3.5 MHz abdominal ultrasound probe manufactured by Interson Corporation for 2-D ultrasound (http://www.interson.com).

Our system is based on Google's Android platform which we chose because it is fully open source and capable of utilizing all the modern features provided by cellular operators. We have tested the system in two configurations: a) running on HTC G1 mobile phone and b) running in an emulator environment on Asus EEE 1000HE netbook computer.

Since USB host mode is not enabled on the conventional HTC G1 phone, it was not possible to connect the USB ultrasound probe to the mobile phone. For this reason we have designed a frame-grabber software module, which is responsible for capturing the raw data from the ultrasound probe and sending it to the G1 phone over short-range wireless network. We've used the same frame-grabber interface when we tested the system in an Android emulation environment running on Asus netbook. Android-powered netbooks are expected to appear in the nearest future and we envision our system running natively on those computers, getting the ultrasound data directly from the available USB port.

Although they have made great progress in the recent years, the cellular data channels available today are still limited when compared to broad-band Wi-Fi alternatives. Even HSDPA, commonly referred to as 3.5G, provides 14.0 Mbps downlink under optimal conditions and HSUPA, which is the uplink component of 3.5G, provides an uplink of up to 5.76 Mbps. This is especially true in developing nations where available cellular services tend to lag behind the cutting edge technologies available in the developed world. This is important because medical imaging devices are often known for generating large quantities of data.

For this reason it is important that the mobile console provides a buffering zone between the actual sensor and the processing station. Even if the connection channel is low-speed and/or unreliable, given enough storage space, the mobile console will eventually succeed to send the data to the processing station once the connection becomes stable.

An alternative scenario might involve a local health worker acquiring large amounts of data from multiple patients and later, when he is back to the local clinic where wi-fi is available, uploading all the accumulated data to the remote station for processing.

**Figure 2:**
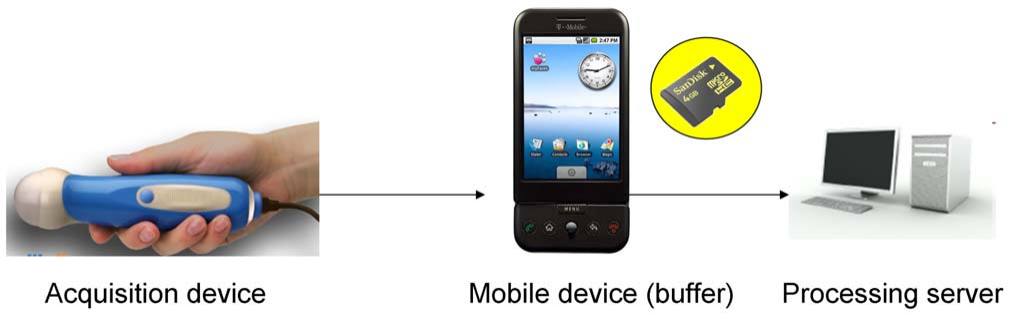
Data Storage Mechanism. The raw data flowing from the mobile device which acts as a storage device. Once a connection is available, the data is being transferred to the server for processing.

Fortunately, the costs of memory have dropped dramatically in the recent years (a 16 gb micro-sd supported by the G1 costs less than $45) so the buffering problem can be efficiently solved; the mobile device (netbook/cellular phone) would accumulate the data on it's internal memory card until connection for uploading this data is available. The raw data flows from the acquisition device, an US probe in our case, to the mobile device which is a mobile phone acting as a storage device, and then transferred to the processing server when the connection is available (Fig. 2).

**Figure 3:**
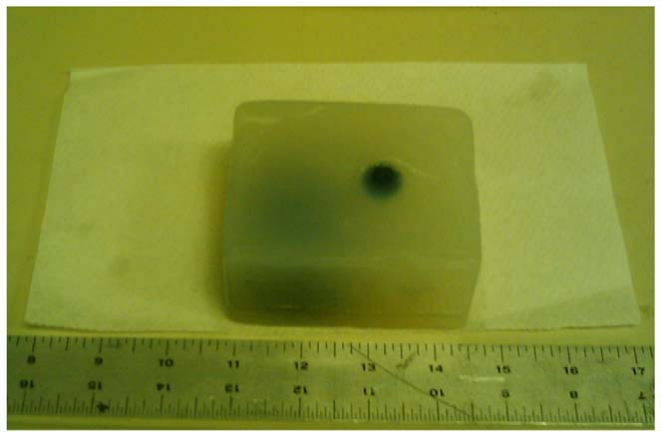
Ultrasound Phantom. Agar based 3.5″x2.75″x2″ box shaped phantom with embedded marble ball, peach pit and two cherry pits. The marble ball and the peach pit can be seen from the image.

To demonstrate a typical scan, we have followed an example from [15] and created an agar based box-shaped phantom, sized 3.5″x2.75″x2″. During the solidifying process, we've embedded a marble ball, a peach pit and two cherry pits inside the phantom to be able to trace those objects in the resulting ultrasound scan (Fig. 3)

Since our purpose was to generate 3-dimensional images, we needed some type of system to provide with positional information. We used a hand held steadily moved probe to avoid the need for a more complex positioning system. It has not escaped our attention that for a truly freehand 3D-US a positioning system is preferable, otherwise the image resolution is extremely low and the image is unusable for clinical purposes. Nevertheless we have intentionally chosen to work around the position information problem since the focus of our work is the feasibility of the overall data acquisition and 3-D processing framework. We provide a brief review of possible alternatives for position and orientation estimation later in this paper.

For performance evaluation, relevant measurements are summarized in Table 1.

**Table 1:** Performances measurements.

Raw data size for a single B-Mode raw image	512 kB
Average raw data transfer for a single B-Mode raw image data (Wi-Fi)	3.9 sec
Volume rendering of 80 slices	28 sec
Snapshot generation for angular resolution of 10 degrees, yielding 36 projections per rotation axis	115 sec
36 Angular snapshot images transfer back to the mobile console (Wi-Fi)	31 sec

As can be seen from the time measurements, we transfer substantial amounts of raw data over the wireless connection, thus the round-trip time is not real-time. Although it is possible to make our system more efficient by using various data compression and channel quality adaptive algorithms, we'd like to emphasize an important point: *due to the nature of the 3D ultrasound, the need for real-time feedback is removed because no hand-eye coordination is required anymore. The relatively unskilled health worker can acquire the data in a freehand manner and after the remote processing is done, have the complete 3D volume data available for review and diagnostic purposes.*

**Figure 4:**
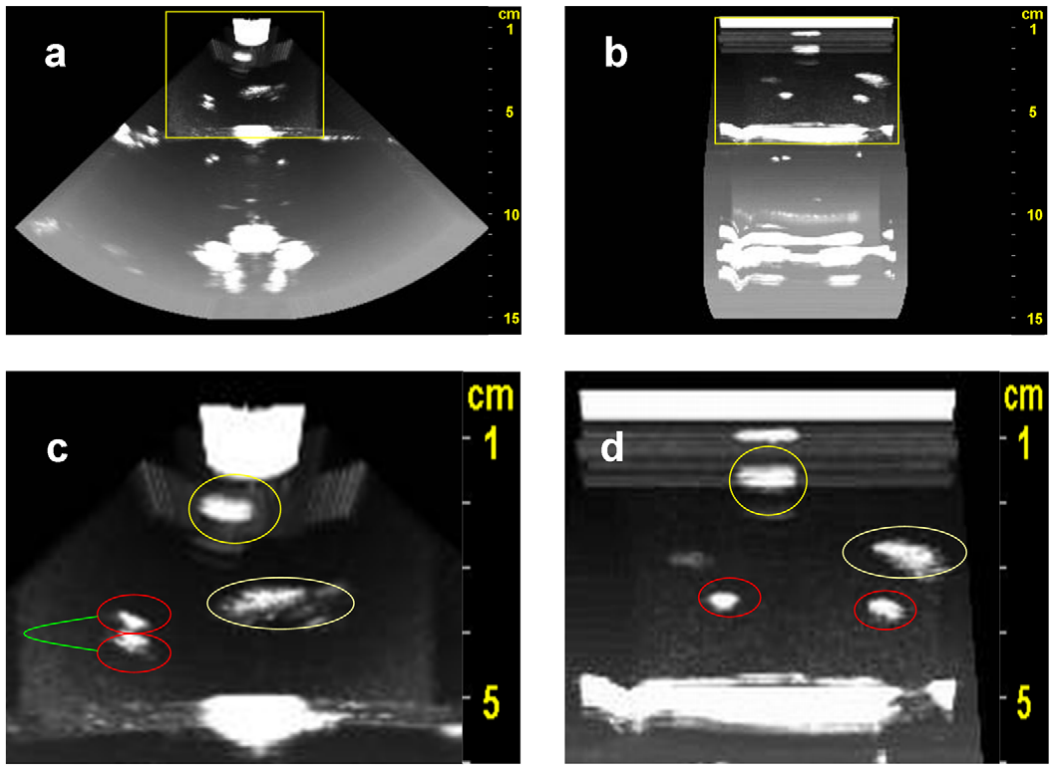
Resulting 3D Volume Visualized. (a) Front projection, axial angle 0°, depth of 15 cm (b) Side projection, axial angle 90°, depth of 15 cm (c) Zoom on ROI from (a): the cherry pits, the peach pit and the marble ball are clearly seen. (d) Zoom on ROI from (b) the cherry pits, the peach pit and the marble ball are clearly seen.

A snapshot of the 3D reconstructed phantom is presented in multiple projection views (Fig. 4a and 4b). The ROI (region of interest) is shown in higher zoom level (Fig. 4c and 4d) where the marble ball can be seen on the top, the peach pit on the right and two cherry pits on the left part of the scan (Fig. 4c)

## Discussion

We've shown in this work the feasibility of performing a 3D ultrasound scan using an inexpensive ultrasound transducer designed for 2-D, a mobile device, a remote processing station and a wireless connection link between them. Acquiring 3D ultrasound data removes the requirement of having a highly trained expert since hand-eye coordination process becomes obsolete. This enables medical workers in developing nations to administer a more accurate diagnosis, effectively saving the lives of people who would have otherwise been misdiagnosed.

It has to be noted that although our system did not incorporate any position information, due to the relatively steady motion of a US transducer by an untrained US user, we managed to get reasonable 3D results, without any positioning device or hand-eye coordination. To provide the health worker with even higher degree of freedom and flexibility during data acquisition we intend to research cost-effective position information mechanisms which can be embedded in our system as a part of our effort to design an affordable and effective medical imaging mechanism for developing nations.

Although our case study focused on US, the implementation of any another medical technology would be identical in its conceptual essence. We chose US since, due to it's mobility and wide availability, it seems like the natural choice of medical diagnostic modality for the developing world. In addition, ultrasound utilizes the available cellular connection as opposed to EIT described in [7] which sends very little data. We expect medical imaging solutions following the paradigm we've demonstrated in this work to appear in the foreseeable future. Constantly lowering mobile devices costs and communication technology advancements will contribute to this process.

An alternative and conceptually similar solution, might include integrating a data acquisition device such as the ultrasound probe used in our case-study with a cellular-phone chip such as, for example Gobi or Snapdragon technologies by Qualcomm (http://www.qctconnect.com/products/snapdragon.html,http://gobianywhere.com/). This solution would include a small display which is capable of displaying the diagnostic information after the remote server has finished processing the raw data. This architecture can be utilized in a consumer device. The possible drawback of such architecture is binding the medical device to a specific cellular technology such as CDMA or GSM. A solution to this problem might include a Bluetooth transmitter in the end device which will send the raw data to any standard cellular phone; most modern phones include Bluetooth capabilities in them. This would expand the possible reach of the technology, since now we can leverage any existing cellular infrastructure.

**Figure 5:**
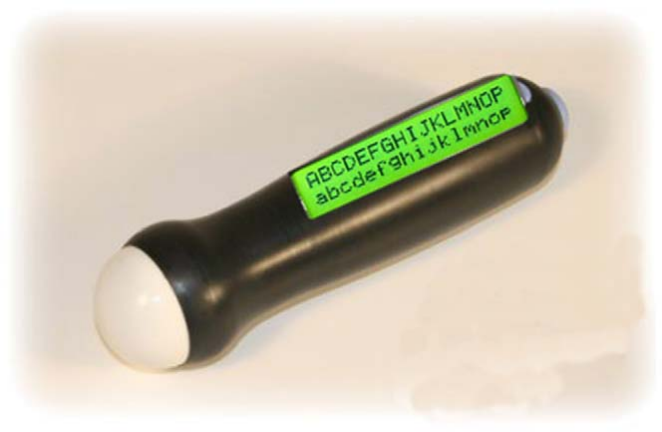
Integrated breast cancer self-examination device for home use.

One such possible device could be used to perform the scan by a health worker or even a home user. The raw data acquired by the Data Acquisition Device would be sent to remote station for processing and a diagnostic result in the form of a text message would be displayed on the LCD line: *“Healthy”* or *“Thorough test is required”* (Fig. 5).

A class of such devices for self-diagnosis is the natural extension of our work and having such a device would enable early detection of diseases, such as cancers or internal bleeding, thus potentially saving the lives of many.

## Materials and Methods

We will describe here the details of the 3-D ultrasound system implemented in this study using the general raw data transfer and data processing algorithm described in Fig. 1. Ultrasound imaging utilizes acoustic waves for the mapping of internal organs and tissues from changes in acoustic impedance between the tissues. Ultrasound works by sending acoustic pulse waves towards the mapped organ and then reconstructing the echoes of those waves into a visual image used for medical diagnosis. Due to the relatively compact size and low power consumption, ultrasound provides an important alternative to other medical imaging modalities such as CT and MRI.

In classic 2D ultrasound, the trained radiologist views the monitor while constructing a mental 3D image of the patient's body. The quality of the diagnostic is heavily biased in the favor of a well-trained radiologist with excellent hand eye coordination and ability to integrate a sequence the 2-D images into a 3-D mental understanding of the image. In 3D ultrasound systems, computer algorithms reconstruct a 3D image from the acquired 2-dimensional images, and therefore simplify the diagnostics. Since the reconstruction engine needs to position the 2D image in the 3D volume, in addition to the image data itself, the exact position and orientation of the US probe are required for each 2D image taken. Several approaches to estimating position and orientation are described at the end of the materials and methods section.

On a highly abstract level, any typical Ultrasound Imaging System includes 4 primary components: a) Transducer – a unit which emits and receives the acoustic waves and records the correlation between them, b) Control unit - used to control the operation of the transducer, c) Processing unit – which converts the raw data acquired by the transducer into a human usable form, usually a visual image, and d) Imaging – the final stage of the ultrasound scan chain where the visual image is being displayed on the monitor for diagnostic purposes.

**Figure 6:**
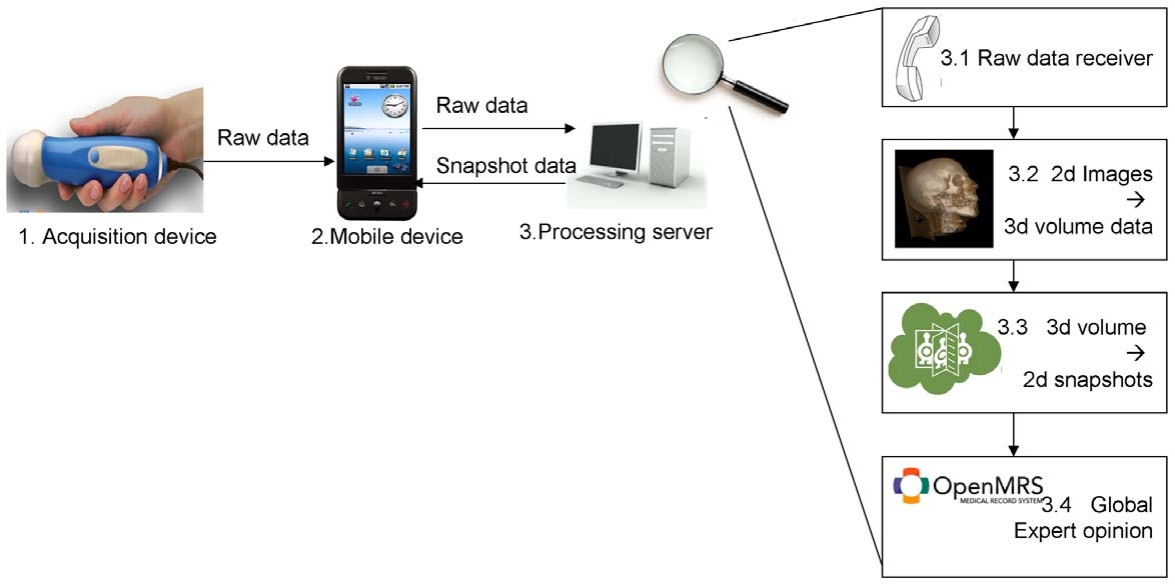
Data flow. The raw data flows from the hardware acquisition device to the mobile console which acts as a storage and communication conduit. Once the data is processed on the server, the results are transferred back to the console for review and diagnosis.

The detailed step-by-step implementation of our wireless 3-D ultrasound algorithm as illustrated (Fig. 6):

1. The raw data arrives from the ultrasound probe.
2. The data arrives to the mobile device which stores the information on its internal memory card until a reliable connection channel becomes available.
3. Every once in a while (frequency can be configured trading-off responsiveness vs. battery life) the mobile device tests the available connection in order to detect the right moment to send the data. Once a connection has been established, the data transfer begins to the processing server. The communication protocol between the mobile device and the processing server is based on XML-RPC (http://www.xmlrpc.com/) which in turn is based on the standard HTTP protocol for transport. The data is packaged in a way that supports operating in slow, unreliable connection channels.
  3.1 Once all the raw data arrives to the server, the processing stage can begin. The data is grouped by the slice number it belongs to.
  3.2 In this stage a stack of parallel slices is being turned into a volume data-set for later manipulation. This can be achieved using the “DICOM Volume Render” open source software module by Mark Wyszomierski which is based on the popular graphics engine VTK.
**D**igital **I**maging and **Co**mmunications in **M**edicine (**DICOM**) is a standard for handling, storing, printing, and transmitting information in medical imaging. In addition to the raw image data, DICOM format enables incorporation of various meta-parameters for example, in our case slice sickness, slice number e.t.c. To design and build a quick prototype, we have decided to skip the direct generation of DICOM files, a process which might easily become mundane. Instead we have downloaded an existing 3D Ultrasound and simply replaced the raw image data with our data, in addition to modifying the relevant parameters.
    Once this process of generating the DICOM files is complete, the renderer can process the stack of 2d images in DICOM format and create a volumetric data-set which is later snapshot to generate multiple view projects for 3d visualization.
  3.3 Once the volumetric data-set has been created, it is projected in multiple directions to create the effect of 3D viewing on the mobile device. Given a high enough angular resolution, the effect is close to a full 3D manipulation in the commonly used axial, sagittal and coronal planes. It's worth noting that recent technological advances in mobile devices, specifically in CPU power and graphic processing abilities, already allow many cellular phones to perform 3D rendering on the mobile unit itself, as demonstrated by [16]. The trade-off decision of battery life vs. visualization power will have to be taken into account by any application designer in the mobile medical imaging field. We've decided to benefit from both worlds by enabling limited 3D visualization by pre-computed projections.
    Once the projections have been generated they are saved as jpeg formatted images which are sent back to the mobile device, again using the XML-RPC over HTTP protocol. By using jpeg images as opposed to sending the volumetric data and rendering the data on the mobile device, we engage only the image-displaying capability of the mobile device as opposed to it's power-hungry 3D engine, thus saving precious battery life.
    One minute, yet important aspect of communication has to be noted: due to the nature of a mobile device, its IP address is highly unstable. The cellular network might decide at a certain point that the IP address of a certain mobile device has to be changed to a different one. This makes it difficult for the server to contact the mobile console to notify it that the processing was completed and results are pending. Even if the mobile console sends its ID to the server, in the time period between the raw-data transmission to the termination of the processing phase, the IP address might have been changed. For this reason we've implemented a console-driven polling mechanism. Once the raw data has been sent, every once in a while, the mobile console polls the server if the results are ready. If they are, the console makes a request for them. The frequency of the polling procedure is a system parameter which can be configured to trade off responsiveness vs. battery life. We've found that the value of T_period_  = 30 seconds provides reasonable results.
  3.4 Global Expert Opinion: to provide an optional expert opinion to the remote health worker, we have integrated our system with OpenMRS® (http://www.openmrs.org), a popular open source medical records system. Once the raw data has been reconstructed and 3D images are available, the processed images are being displayed in a “pending” queue in OpenMRS. After a medical expert reviews the data and adds his comments, the result is sent to the mobile console for display. Because we adapted the concept of distributing the components of the imaging system, the expert reviewing the diagnostic images can be at any geographic location, unrelated to the location of the health worker or the processing station. What this means is that a local health worker in rural Uganda can perform a scan that is being processed in data servers in India and an expert radiologist from the U.S. provides a diagnostic opinion which is sent with the results back to the local health worker effectively in real-time.

An important aspect of any 3D ultrasound system concerns position and orientation information. During the process of 3-dimensional image reconstruction, every surface element (pixel) from the 2-dimensional images is mapped to a volume element (voxel) in the 3D reconstructed volume. To perform such a mapping accurately, the reconstruction algorithm needs to know the precise position and orientation of the ultrasound probe at the moment of the 2D image acquisition. There are several techniques to this end. Mercier et al. review common technologies for medical instruments tracking [17]. Electro-magnetic and optical technologies for ultrasound probe tracking are the most popular. While those approaches provide good accuracy, they are also relatively bulky and expensive. Since we are working with the needs of developing countries in mind, we want to emphasize more mobile, cost-effective solutions. Abdul Rahni et al. have studied the possible usage of Micro Electro-Mechanical Systems (MEMS) based approach to estimate position and orientation in 3D [18]. In their study, the authors have used an Internal Measurement Unit (IMU) which included an accelerometer and a gyroscope. The advantage of this approach is its simplicity – no external camera or receiver is needed, as in the electromagnetic/optical technology case. The raw physical measurements (acceleration, angular velocity and static orientation) are read from the IMU and processed to calculate the absolute 3D position and orientation. By adding redundant sensors, it is possible to compensate for some of the numeric errors inherent to the process. Another work that has caught our attention has used a conventional digital camera for position and orientation estimation [19]. During the data acquisition process, in addition to the US data, a video clip focusing on the ultrasound probe is captured. After the acquisition process is over, the position and orientation information are extracted by applying machine vision algorithms to the acquired video stream. By using a conventional digital camera, which often comes as an integral part of any modern cell-phone, it is possible to build a low-cost, ultra-mobile 3D position mechanism.

 We believe that the approaches presented in [18], [19] can be used as a basis for a cost-effective, mobile position and orientation estimation mechanism which are required by a 3D reconstruction algorithm and we intend to explore those research directions. Since the primary focus of our current work was to illustrate the concept of the overall data acquisition and 3-D processing framework, we've decided to relax the freehand requirement and work around the 3D positioning issue by steadily moving the US probe in a straight line during the data acquisition stage. By sticking to the straight line trajectory, we were able to use a more straightforward reconstruction algorithm since it could simply stack the 2D images one next to each other and still get a 3D images of reasonable quality. In the future we intend to develop relevant variants of the techniques described in [18], [19].
